# Protective Role of Vitamin D Supplementation in Proliferative Diabetic Retinopathy in a Type 1 Diabetes Premenopausal Woman: A Case Report from the region of Dobrogea, Romania

**DOI:** 10.22336/rjo.2025.72

**Published:** 2025

**Authors:** Nejla Dervis, Sanda Jurja, Cristina Cioti, Ana-Maria Stoica

**Affiliations:** 1 Department of Diabetes, Ovidius University, Constanţa, Romania; 2 Department of Ophthalmology, “Sf. Apostol Andrei” Emergency County Hospital, Constanţa, Romania; 3 Department of Internal Medicine, “Sf. Apostol Andrei” Emergency County Hospital, Constanţa, Romania

**Keywords:** Vitamin D, diabetic retinopathy, retinal neovascularization, type 1 diabetes, CGM = Continuous Glucose Monitoring, DR = Diabetic Retinopathy, HbA1c = Hemoglobin A1c, HIF = Hypoxia-Inducible Factor, OCT = Optical Coherence Tomography, PDR = Proliferative Diabetic Retinopathy, PRN = Pro re nata (as needed), PRP = Panretinal Photocoagulation, VA = Visual Acuity, VD = Vitamin D, VEGF = Vascular Endothelial Growth Factor

## Abstract

**Objectives:**

To present the case of a premenopausal woman with long-standing type 1 diabetes mellitus and diabetic retinopathy, in whom severe vitamin D deficiency was associated with poor metabolic control and depressive symptoms, and whose ocular and systemic condition spectacularly improved after vitamin D supplementation.

**Material and methods:**

We describe the clinical history, laboratory findings, and ophthalmological assessments of a 42-year-old woman from an urban area of Dobrogea, Romania. The patient had a 28-year history of type 1 diabetes mellitus complicated with proliferative diabetic retinopathy, early-stage chronic kidney disease, and diabetic polyneuropathy. Initial laboratory evaluation indicated severe vitamin D deficiency (8 ng/mL), marked glycemic variability (documented by continuous glucose monitoring), and microalbuminuria.

**Results:**

Following six months of vitamin D supplementation, serum levels increased to 40 ng/mL, depressive symptoms improved, and the patient became more adherent to nutritional therapy. Ophthalmological follow-up: Optical Coherence Tomography (OCT) retinal findings showed stabilization of retinal neovascularization.

**Discussion:**

The distinctiveness of this case arises from the coexistence of multiple risk factors, including long-duration type 1 diabetes with microvascular involvement, pronounced vitamin D deficiency despite favorable sun exposure conditions, premenopausal status under HRT, and retinal changes objectively documented before and after supplementation.

**Conclusions:**

This case supports the hypothesis that vitamin D may have a protective role against retinal neovascularization in diabetic retinopathy, possibly by reducing inflammation and angiogenesis trend, and indirectly by improving metabolic control.

## Introduction

Epidemiological data predict a substantial rise in the prevalence of diabetes and its most common complication, diabetic retinopathy (DR), for which effective preventive and therapeutic strategies are still lacking [1-4]. Hypovitaminosis D is frequently observed in patients with diabetes, and vitamin D (VD) has been recognized to have vascular protective properties [5]. Accordingly, numerous studies have examined the association between VD deficiency and DR, including its severity and progression [6-8]. In contrast, the impact of VD supplementation on the natural history of diabetes remains unclear [8].

## Case presentation

A 42-year-old premenopausal woman from an urban area, undergoing hormone replacement therapy (HRT) with estrogen and progesterone, presented to the Ophthalmology Department for ophthalmic follow-up. She had a 28-year history of type 1 diabetes mellitus, diagnosed at the age of 14, complicated by proliferative diabetic retinopathy (PDR) and diabetic polyneuropathy. Her medical history was also notable for a pregnancy complicated by fetal macrosomia. The family history was positive for both type 1 and type 2 diabetes mellitus. The patient was an active smoker and exhibited suboptimal adherence to nutritional therapy. This pattern appeared to be associated with her ongoing anxiety and the depressive symptoms she has been experiencing for approximately three months.

Laboratory assessment revealed:

- Marked glycemic variability on continuous glucose monitoring (CGM) with glycemic values between 200 and 400 mg/dl;
- Microalbuminuria: **30-300 mg/g;**
- Severe vitamin D deficiency: 8 ng/mL;

The patient was managed with oral vitamin D supplementation in conjunction with panretinal photocoagulation (PRP) and intravitreal administration of the anti-VEGF agent aflibercept, in accordance with current therapeutic guidelines. The patient was provided with reinforcement of nutritional therapy principles, instruction on appropriate insulin injection techniques, and guidance on adjusting insulin doses in relation to glycemic values. Furthermore, psychological counseling was considered advisable to facilitate smoking cessation and to address symptoms of anxiety.

After six months of supplementation, the vitamin D serum level increased to 40 ng/mL, depressive manifestations showed a favorable evolution, and the patient reported better adherence to nutritional and insulin therapy (**Table 1**).

**Table 1:** Patient parameters before and after vitamin D supplementation

Parameter	Before Vitamin D Supplementation	After 6 Months of Supplementation
Vitamin D (ng/mL)	8	40
HbA1c (%)	14	7.7
Mean CGM glucose (mg/dL)	355	174
Microalbuminuria	Present	Reduced
Fundus findings	Proliferative DR with neovascularization of the optic disc, retinal hemorrhages (dot-blot and flame-shaped), hard exudates, and soft exudates (cotton-wool spots).	Regression of neovascularization at the optic disc with fibrotic remnants, no new vessels observed—significant reduction of retinal hemorrhages. Fewer hard exudates, cotton-wool spots largely resolved.
OCT findings	Diabetic macular edema was more frequent, with retinal thickening and cystic spaces, and hyperreflective exudates.	Decrease in diabetic macular edema with reduced retinal thickening. Cystic intraretinal spaces were less prominent.

**Fig. 1:**
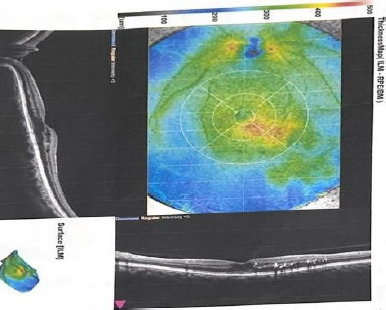
OCT scan of the macula before supplementation - presence of edema/thickness

**Fig. 2:**
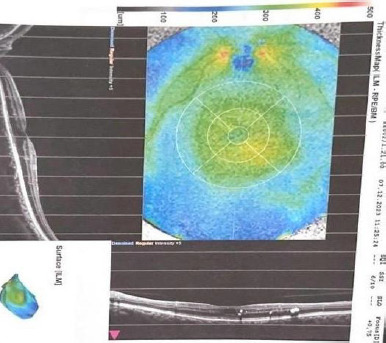
OCT scan of the same region after supplementation - aspect of improvement/stabilization

Ophthalmological evaluation before supplementation revealed proliferative diabetic retinopathy with neovascularization of the optic disc, retinal hemorrhages (dot-blot and flame-shaped), hard exudates, and soft exudates (cotton-wool spots). OCT revealed diabetic macular edema with retinal thickening and cystic intraretinal spaces, accompanied by hyperreflective exudates, as shown in **Fig. 1**. At the six-month follow-up, the fundus and OCT findings indicated a favorable therapeutic response, with regression of neovascularization and improvement of diabetic macular edema, as highlighted in **Fig. 2**.

## Discussion

This case highlighted once more the potential multifactorial role of vitamin D supplementation in a patient with long-standing type 1 diabetes mellitus complicated by proliferative diabetic retinopathy (PDR).

The main findings of our case include the severe baseline vitamin D deficiency in a premenopausal woman, despite her residence in a sunny region (Dobrogea), and the favorable evolution of both glycemic and ophthalmological status, with stabilization of retinal neovascularization following supplementation.

It is important to note that the patient was in the premenopausal period and was receiving HRT with estrogen and progesterone, variables that potentially affect metabolic control and vascular integrity.

Vitamin D has been shown to influence angiogenesis by downregulating vascular endothelial growth factor (VEGF) expression and reducing retinal inflammation [9-11].

Experimental studies suggest that calcitriol, the active metabolite of vitamin D, inhibits retinal neovascularization, likely through effects on hypoxia-inducible factor (HIF) activity [10]. Lower circulating levels of vitamin D have been reported to be associated with a higher risk and greater severity of DR, most notably in type 1 diabetes [12].

In the present case, the patient’s depressive symptoms and poor nutritional compliance might have exacerbated glycemic variability and microvascular damage. Vitamin D therapy appears to have contributed to improvements in mood and metabolic homeostasis, as also suggested by previous studies that correlated vitamin D status with psychological well-being and glucose regulation [13,14].

The stabilization of PDR documented on follow-up fundus and OCT evaluations suggests that vitamin D could have exerted a direct and/or indirect protective influence on the retinal vasculature. The concomitant use of HRT introduces an additional variable, as estrogen and progesterone also modulate vascular and inflammatory pathways, which could potentially converge with the effects of vitamin D.

The distinctiveness of this case arises from the coexistence of multiple risk factors, including long-duration type 1 diabetes with microvascular involvement, pronounced vitamin D deficiency despite favorable sun exposure conditions, premenopausal status under HRT, and retinal changes objectively documented before and after supplementation.

While a causal relationship cannot be firmly established from a single case report, the temporal relationship and supportive findings in the literature provide a reasonable basis for considering vitamin D status in the management strategy for advanced diabetic retinopathy. Considering the association of HRT with reducing pre- and/or menopausal medical risks, supplementary research is required to determine the most beneficial balance of hormones/vitamin D as a goal to achieve, aiming to delay as much as possible the progression of ocular and other diabetes complications.

## Conclusions

Vitamin D administration in a case of advanced diabetic retinopathy with significant hypovitaminosis was correlated with enhanced systemic and ophthalmic outcomes. Although causality cannot be determined from a single case, the findings align with accumulating evidence supporting a potential protective effect of vitamin D in diabetic retinopathy. Continued studies are justified.
